# Special Southern Number

**Published:** 1911-01

**Authors:** 


### Special Southern Number.

The January issue of the *American Journal of Surgery* will be composed entirely of original contributions from the pens of well known Southern surgeons. Among those to appear, we would mention:

"Pyuria," by Howard A. Kelly, M. D., Baltimore, Md.

"Transfusion of the Blood, Its Indication and Technic," by J. Shelton Horsley, M. D., Richmond, Va.

"Tumors of the Lower Jaw, the Form Most Frequently Found in the Negro," by Willis F. Westmoreland, M. D., Atlanta, Ga.

"Pylorospasm," by Stuart McGuire, M. D., Richmond, Va.

"Prevention of Immediate Post-Operation Pain by Quinine Injections," by Drs. V. and V. W. Pleth, Seguin, Texas.

"The Importance of Educating the Public in Regard to Cancer," by Southgate Leigh, M. D., Norfolk, Va.

"Aerogenes Infections," by George R. White, M. D., Richmond, Va.

"Stricture of the Rectum, Complicating Fistulae," by C. S. Venable, M. D., San Antonio, Texas.

"Gastric Symptoms from a Surgical Viewpoint," by Louis Frank, M. D., Louisville, Ky.

Dr. Edgar D. Capps, of Fort Worth, Texas, and H. Berlin, M. D., of Chattanooga, Tenn., will also contribute original articles to this number.

